# Diffuse chorioretinal atrophy after a single standard low- dose intravitreal melphalan injection in a child with retinoblastoma: a case report

**DOI:** 10.1186/s12886-016-0204-6

**Published:** 2016-03-15

**Authors:** An- Ning Chao, Ling-Yuh Kao, Laura Liu, Nan-Kai Wang

**Affiliations:** Department of Ophthalmology, Chang Gung Memorial Hospital and Chang Gung University, College of Medicine, Fu-Hsin Street, Kweishan Taoyuan, 3305 Taiwan

**Keywords:** Intravitreal injection, Retinoblastoma, Melphalan, Toxicity, Retina

## Abstract

**Background:**

Controlling retinoblastoma with seeding is challenging despite advances in treatment modalities. Intravitreal melphalan is an alternative to external beam radiation or enucleation for recurrent or refractory vitreous seeds. Significant ocular side effects following intravitreal melphalan injections are uncommon. Complications have been reported in eyes receiving higher concentrations of melphalan and repetitive injections. We report a case in which diffuse chorioretinal atrophy was developed at the injection site after a single, standard low-dose intravitreal melphalan injection.

**Case presentation:**

A 12-month-old female child without a family history of retinoblastoma presented with unilateral group C retinoblastoma in her right eye. A solitary tumour with retinal breaks on the tumour surface, and vitreous seeds overlying the tumour were observed at the 8 o’clock position of the retina. After two cycles of intra-arterial chemotherapy with melphalan, the main tumour displayed significant regression, but the vitreous seeds overlying the main tumour were still active. Because of the persistence of vitreous seeds and the inadequate response to intra-arterial melphalan treatment, intravitreal melphalan (8 μg in 0.05 mL) was injected using a 32-gauge needle 2.5 mm from the 5 o’clock position of the limbus, the meridian opposite to the vitreous seeds. After 1 month, the retina around the injection site demonstrated diffuse retinal pigment epithelium alterations with dense hard exudates. Although the main retinal mass, and vitreous seeds resolved, the hard exudates persisted for more than 2 years after the single low-dose melphalan injection.

**Conclusions:**

Intravitreal melphalan injections should be cautiously used for eyes with refractory seeds, particularly when multiple injections are required to control retinoblastoma seeds. Dose- related retinal toxicity could occur in pre-treated eyes even when a relatively low standard dose is used. Such patients should be followed up closely to monitor the treatment response and to assess potential delayed toxicity.

## Background

Eye preservation and tumour control in patients with retinoblastoma with seeding are challenging despite advances in treatment modalities. Treatment modalities for vitreous seeding include external beam radiotherapy, brachytherapy, systemic chemotherapy, intra-arterial chemotherapy, intravitreal chemotherapy or a combination of these therapies [1–3]. Intravitreal melphalan is an alternative to external beam radiotherapy or enucleation for recurrent or refractory vitreous seeds because it can achieve a globe salvage rate of 87–100 % [2, 3]. Although significant ocular side effects following intravitreal melphalan injections are uncommon, iris atrophy, chorioretinal atrophy, vitreous haemorrhage and retinal detachment have been reported. These complications are more common in eyes receiving a higher concentration of melphalan and repetitive injections [4, 5]. Here, we report a case in which diffuse chorioretinal atrophy was developed at the injection site after a single- low dose (8 μg) intravitreal melphalan injection that successfully controlled vitreous seeding.

## Case presentation

**Fig. 1:**
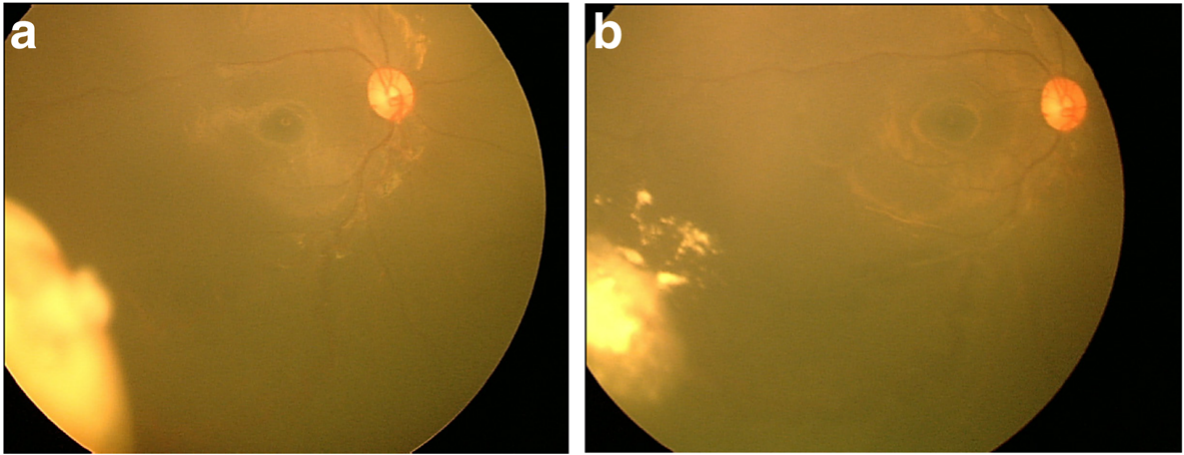
**a** Initial presentation: a solitary tumor at the inferior temporal retina with seeds overlying the tumour (**b**) The main tumor displayed significant regression, but active vitreous seeds persisted after two cycles of intra-arterial melphalan chemotherapy

**Fig. 2:**
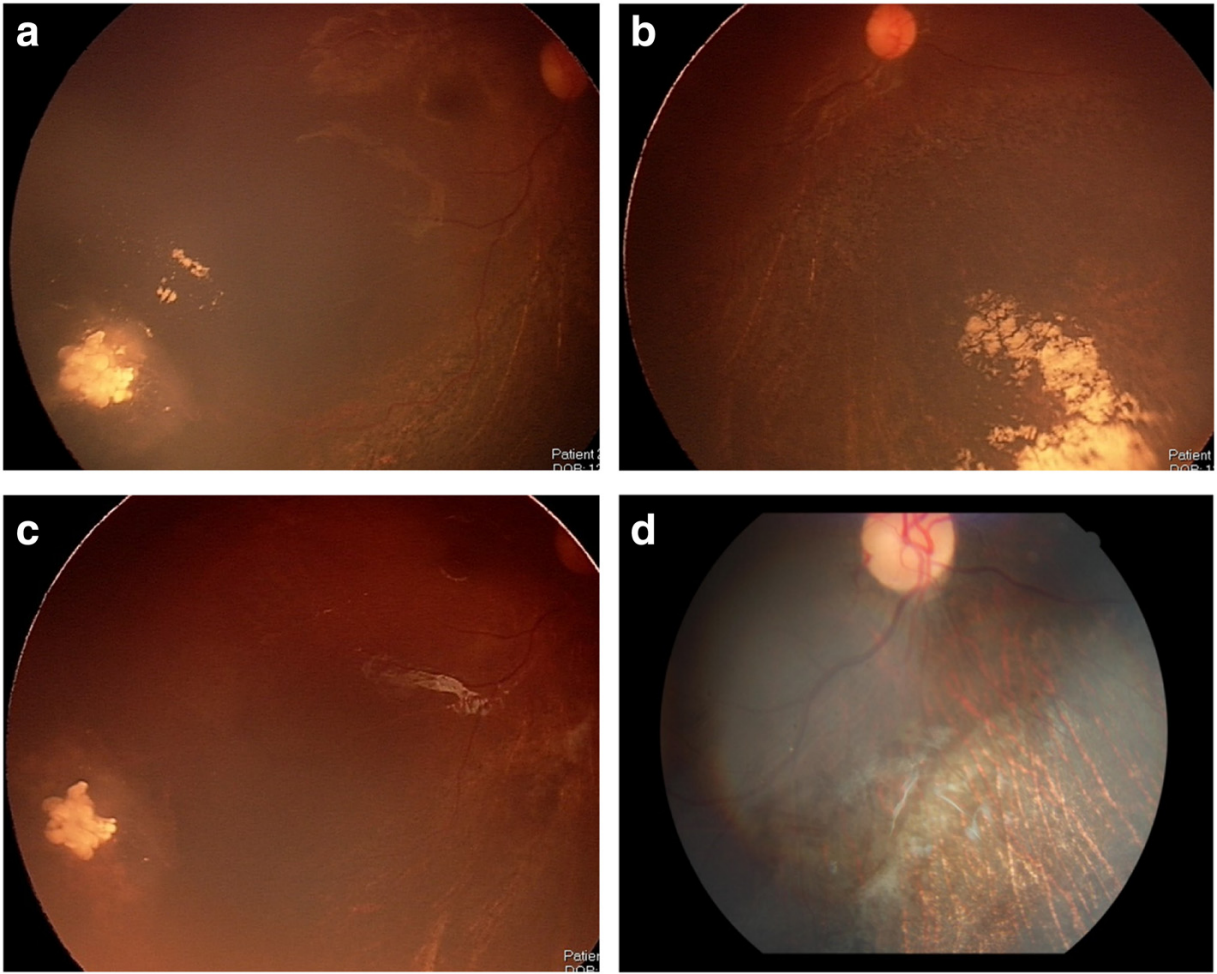
**a** The main retinal tumour at the inferior temporal retina exhibited further regression. **b** Diffuse retinal pigment epithelial alterations and exudates were found at the inferior nasal retina where intravitreal melphalan was injected. **c** and **d** At the 3- year follow-up, the main tumor displayed calcified regression with partial resolved exudates and chorioretinal atrophy

A 12-month-old female without a family history of retinoblastoma presented with unilateral group C retinoblastoma in her right eye. A solitary tumour with retinal breaks on the tumour surface was observed at the 8 o’clock of the retina. Vitreous seeds overlying the tumor were also observed (Fig. 1a). After two cycles of intra-arterial chemotherapy with 5 mg melphalan (Alkeran, GlaxoSmithKline, Italy), the main tumour displayed significant regression but the vitreous seeds overlying the tumour were still active (Fig. 1b). Because of the persistence of vitreous seeds and the inadequate response to intra-arterial melphalan treatment, intravitreal melphalan (8 μg in 0.05 mL) was injected using a 32- gauge needle 2.5 mm from the 5 o’clock position of the limbus, the meridian opposite to the vitreous seeds. Cryotherapy was applied at the injection site, and the eye was moved in all directions for several times to improve drug distribution as previously described [6]. The main retinal mass exhibited further regression, and all vitreous seeds were resolved without further treatment and displayed no recurrence. However, 1 month after the intravitreal injection, the retina around the injection site demonstrated diffuse retinal pigment epithelium alterations with dense hard exudates (Fig. 2a and  b). At the 3-year follow-up, her best corrected vision is 20/60, the exudates partially resolved but diffuse chorioretinal atrophy persisted after the single low-dose of melphalan injection (Fig. 2c and d).

## Discussion

Recurrent or refractory seeds are avascular, and resistant to conventional chemotherapy because of the hypoxic environment of the vitreous seeds. Hence, Intravitreal chemotherapy is an alternative to external beam radiotherapy or enucleation for such seeds. Intra-arterial chemotherapy and direct injection of chemotherapeutic agents, or a combination of these therapies deliver higher chemotherapy drug concentrations fort vitreous seeding treatment, and have less systemic toxicity. The main concerns of intravitreal chemotherapy are tumour spread from the needle tract, and other potential side effects such as endophthalmitis, vitreous haemorrhage and retinal toxicity from chemotherapeutic agents. Melphalan is a cytotoxic alkylating agent that inhibits both DNA and RNA synthesis. It was originally used to treat multiple myeloma and ovarian cancer. Intravitreal melphalan for vitreous seedings was first introduced in the 1990s by Kaneko and Suzuki [7]. Inomata and Kaneko investigated 12 chemotherapy drugs and found that melphalan had the highest tumoricidal effect [8]. Ueda et al. investigated the optimal dose of melphalan on rabbit retina and concluded that intravitreal 10 μg melphalan did not cause histological or electroretinogram (ERG) changes, and that moderate ERG changes were observed after an injection of 20 μg melphalan [9]. Initially, they injected melphalan at a dose of 8 μg; subsequently, the standard dose was increased to 16 μg, and 24 μg was employed for eyes refractory to standard dose after 2008. The seeds exhibited regression after a median of 3.9 injections (range, 1–25 injections). Several recent studies have reported that intravitreal melphalan at doses of 20–30 μg is safe and efficient [2, 3, 5] Shields et al. also demonstrated that vitreous seeds were controlled after a mean of four injections [5].

A systematic review displayed that significant ocular side effects including extraocular extension, retinal detachment, iris atrophy, chorioretinal atrophy, vitreous haemorrhage and retinal detachment following intravitreal melphalan injections (8–30 μg) were uncommon. In the studies included in this review, 1287 injections were administered to 306 eyes, 0.01 % (3/261) of patients developed iris atrophy, 0.07 % (2/295) experienced with retinal detachment, and 0.08 % (2/261) developed chorioretinal atrophy [4]. Suzuki et al. administered 1067 intravitreal melphalan injections to 264 eyes of 250 patients (reported as the largest cohort) between 1990 and 2011. They reported a subconjunctival tumor with extraocular extension in only one eye. Additionally, they reported diffuse chorioretinal atrophy in two eyes several months after three intravitreal injections; of these eyes, one received external beam radiotherapy and one received intra-arterial chemotherapy [7]. Munier et al. administered 122 injections to 23 eyes with vitreous seeds weekly, of which 43 % developed salt and pepper retinopathy at injection sites [2]. They demonstrated that complications were more common in eyes receiving a higher concentration of melphalan and repetitive injections. Moreover, they proposed that the risk of intraocular toxicity could be minimized by injecting melphalan at doses ≤30 μg.

In our case, the unilateral solitary retinoblastoma with vitreous seeds was initially treated with two monthly cycles of intra-arterial melphalan (5 mg); the main tumor exhibited significant regression but the vitreous seeds were still active. To treat seeds refractory to inta-arterial melphalan, we injected 8 μg intravitreal melphalan. Additionally, we followed the recommended guidelines for an intravitreal melphalan injection. However, in our case grade 2 retinal toxicity was developed after a single intravitreal melphalan injection with a dose of 8 μg, a relatively low dose [2]. We believed that diffuse chorioretinal atrophy at the injection was due to intravitreal injection because no signs of retinal toxicity were observed after two cycles of intra-arterial chemotherapy. This adverse drug reaction occured in this eye possibly because it was more vulnerable after intra-arterial chemotherapy.

Intravitreal chemotherapy can achieve a considerably improved eye salvage rate; therefore, we expect intravitreal melphalan to be more commonly used as concurrent or adjunctive treatment for globe salvage. Although significant ocular complications following intravitreal melphalan are uncommon, unexpected retinal toxicity could occur even when the standard dose and a careful technique are employed particularly, in eyes received multiple treatments.

## Conclusions

Intravitreal melphalan injections should be cautiously used when multiple injections are required to control recurrent or persistent retinoblastoma seeds after treatment with systemic or intra-arterial chemotherapy. Although significant ocular side effects are uncommon, and extraocular extension is rare, clinicians should be aware of its cumulative effects, and its narrow therapeutic window. Dose related retinal toxicity could occur in patients with pre-treated eyes even with a relatively low standard dose. Such patients must be followed up closely to monitor the treatment response and assess potential delayed toxicity.

## Consent

Written informed consent was obtained from the father of this patient for publication of this Case report and any accompanying images. A copy of the written consent is available for review by the Editor of this journal.
